# An analysis of the influence of deep neural network (DNN) topology in bottleneck feature based language recognition

**DOI:** 10.1371/journal.pone.0182580

**Published:** 2017-08-10

**Authors:** Alicia Lozano-Diez, Ruben Zazo, Doroteo T. Toledano, Joaquin Gonzalez-Rodriguez

**Affiliations:** Audias-UAM, Universidad Autonoma de Madrid, Madrid, Spain; Nanjing University, CHINA

## Abstract

Language recognition systems based on bottleneck features have recently become the state-of-the-art in this research field, showing its success in the last Language Recognition Evaluation (LRE 2015) organized by NIST (U.S. National Institute of Standards and Technology). This type of system is based on a deep neural network (DNN) trained to discriminate between phonetic units, i.e. trained for the task of automatic speech recognition (ASR). This DNN aims to compress information in one of its layers, known as bottleneck (BN) layer, which is used to obtain a new frame representation of the audio signal. This representation has been proven to be useful for the task of language identification (LID). Thus, bottleneck features are used as input to the language recognition system, instead of a classical parameterization of the signal based on cepstral feature vectors such as MFCCs (Mel Frequency Cepstral Coefficients). Despite the success of this approach in language recognition, there is a lack of studies analyzing in a systematic way how the topology of the DNN influences the performance of bottleneck feature-based language recognition systems. In this work, we try to fill-in this gap, analyzing language recognition results with different topologies for the DNN used to extract the bottleneck features, comparing them and against a reference system based on a more classical cepstral representation of the input signal with a total variability model. This way, we obtain useful knowledge about how the DNN configuration influences bottleneck feature-based language recognition systems performance.

## Introduction

The task of Language Recognition or Language Identification (LID) is defined as the task of identifying the language spoken in a given audio segment [1]. Automatic systems for LID aim to perform this task automatically, learning from a given dataset the necessary parameters to identify new spoken data.

There are multiple applications of this technology as, for example, call centers that need to classify a call according to the language spoken, speech processing systems that deal with multilingual inputs, multimedia content indexing, or security applications such as tracking people depending on their language or accent.

Moreover, language recognition shares important modules with many other systems from closely related fields like speaker recognition (the task of identifying the person who is speaking in a given utterance), speech recognition (transcribe audio segments), or, in general, speech signal processing. Furthermore, not just the speech signal processing research area is involved, but also techniques from the machine learning field. In fact, the successful application and adaptation of machine learning tools is one of the main lines of research in language recognition nowadays.

### NIST language recognition evaluations

Research in the field of language recognition has been driven to a large extent by the Language Recognition Evaluation (LRE) series organized by NIST (U.S. National Institute of Standards and Technology) approximately every two years since 1996 and up to 2015. This technology evaluations provide a common framework (making data available to all participants) to evaluate a given recognition task. Each participant sends results to the organization, which later provides comparative results, and final conclusions are shared during a workshop.

Each evaluation differs in the specific tasks that participants have to address, such as dealing with different test duration, various languages, channel variability or noise conditions.

The last two evaluations (corresponding to 2011 and 2015) have focused on the task of identifying similar languages (dialects or highly related languages), and, especially, testing short audio segments (less than 10 seconds) which has become a main concern nowadays. In particular, the last NIST LRE 2015 divided languages according to clusters of similar languages, which will be the task addressed in this work.

As we already mentioned before, machine learning techniques conform a big research line in the field of language recognition. In this context, two of the evaluations organized by NIST in 2014 and 2015 [2, 3], known as *i-vector challenges*, skipped all the audio processing up to the i-vector (a fixed length vector representation of a given utterance which contains information useful for the target task, described in detail in Section Language Recognition: the i-vector approach). This way, participants addressed speaker and language recognition tasks, but establishing the i-vector as starting point instead of audio files, which allowed participants to focus on the machine learning algorithms for the classification stage, and supported the consolidation of i-vectors as successful models to tackle language recognition.

### State-of-the-art in language recognition

Progress in language recognition has been closely related to speaker recognition. Thus, techniques that were breakthroughs in the speaker recognition field, were adapted and successfully applied to improve LID systems. That is the case of the fixed-length representation of utterances, known as i-vector [4] (described in Section Language Recognition: the i-vector approach), which captures information meant to be useful for the target task. This framework has been the state-of-the-art in language recognition since 2011 [5, 6] until the success of Deep Neural Networks (DNNs), complex machine learning models that try to emulate some aspects of the human brain behavior (learning abstract representations of the input data), which improved notably the ability of automatic systems to model speech signals [7].

Traditionally, language recognition systems have been based on acoustic features such as MFCCs (Mel Frequency Cepstral Coefficients), which are a short-term cepstral representation of the audio signal, in the Mel-frequency domain. These feature vectors, extracted every few milliseconds (typically, 10 ms), are then modeled by Gaussian Mixture Models (GMMs), probabilistic models composed of a weighted sum of Gaussian distributions. In order to compensate the variability present across different utterances from the same language or speaker, Factor Analysis (FA) was introduced. FA is a generative model that aims to disentangle speaker variability from channel variability. Even though FA was a very good theoretical model, speaker and channel variability subspaces were not easy to estimate, especially when datasets used to estimate them did not contain enough variability. Therefore, a new modeling approach known as Total Variability (TV) was introduced, which aims to gather all the variability in the same relatively low dimensional subspace. Using this TV subspace, each utterance is then represented by a fixed-length vector (typically, 400 or 600 dimensional vector), the i-vector. This last approach outperformed FA and has been the state-of-the-art for many years in language and speaker recognition.

More recently, the successful introduction of DNN based approaches in the acoustic modeling of Automatic Speech Recognition (ASR) systems, motivated its application in related fields such as language recognition. In this field, end-to-end DNN approaches (i.e., DNN as the only tool used as classifier given some input features) have not shown relevant success in performance yet. However, the use of DNNs to learn automatically a new representation of the input data has become a breakthrough in the field of language recognition. Thus, the DNN is used as a feature extractor in state-of-the-art LID systems. These feature vectors extracted from the DNN, known as bottleneck (BN) features (described in detail in Section Bottleneck Features for Language Recognition), are frequently used as input to the traditional i-vector pipeline, instead of the typical acoustic parameters (as MFCCs). These language recognition systems based on bottleneck features outperformed notably previous approaches, consolidating its success since the last NIST LRE 2015, and it will be the approach used in this work.

### Motivation

The influence of changes in the topology of the DNN used as bottleneck feature extractor in language recognition has not been analyzed in detail in previous published works [8–11]. Thus, it is not known how variations of the configuration of the DNN might affect the final performance of the language recognition system. For this reason, in this paper we analyze different topologies of the DNN in terms of final language recognition performance, over the same dataset and framework. Feedforward DNNs are trained to automatically learn a transformation that maps an input to an output. Each of the hidden layers is then learning features which help with that discriminative task. It has been seen in different research works [12] that deeper layers learn more abstract features, with more information which help to final classification. In this work, the DNN has a bottleneck layer that compresses the information [13] learnt up to that point by the network. Then, depending on the configuration of the network, and the bottleneck layer in particular, the information captured (and thus, the representation of the recording) will be different. For this reason, the structure and position of the bottleneck layer affects the information captured, which are then used as new feature vectors representing the input data to the LID system.

In particular, in this work, we train a DNN for the task of automatic speech recognition (ASR), which we use as feature extractor, and vary some hyper-parameters involved in the design of it, such as the number of hidden layers or position and size of the bottleneck layer. Then, as we have mentioned, all these hyper-parameters may affect the performance of the system since the representation learnt by different DNN configurations might content different information, more or less useful, for the final task of LID. For example, we explore how the position of the bottleneck layer, either closer to the input layer, or moving towards the output layer, obtaining higher level information each time, influences the final system performance. Finally, we look for the best configuration of the DNN for the target task of language recognition in the setup used in this work.

### Paper organization

The rest of the paper is organized as follows. First, an introduction to DNN is presented in Section Deep Neural Networks and Speech Processing. Second, language recognition systems based on bottleneck features are explained in Section Bottleneck Features for Language Recognition, and the specific framework used in this work is described in Section Experimental Framework. Then, experiments and results developed in this work are analyzed in Section Experiments and Results. Finally, conclusions from this work are drawn and summarized in Section Conclusions.

## Deep neural networks and speech processing

### Deep neural networks description

**Fig 1:**
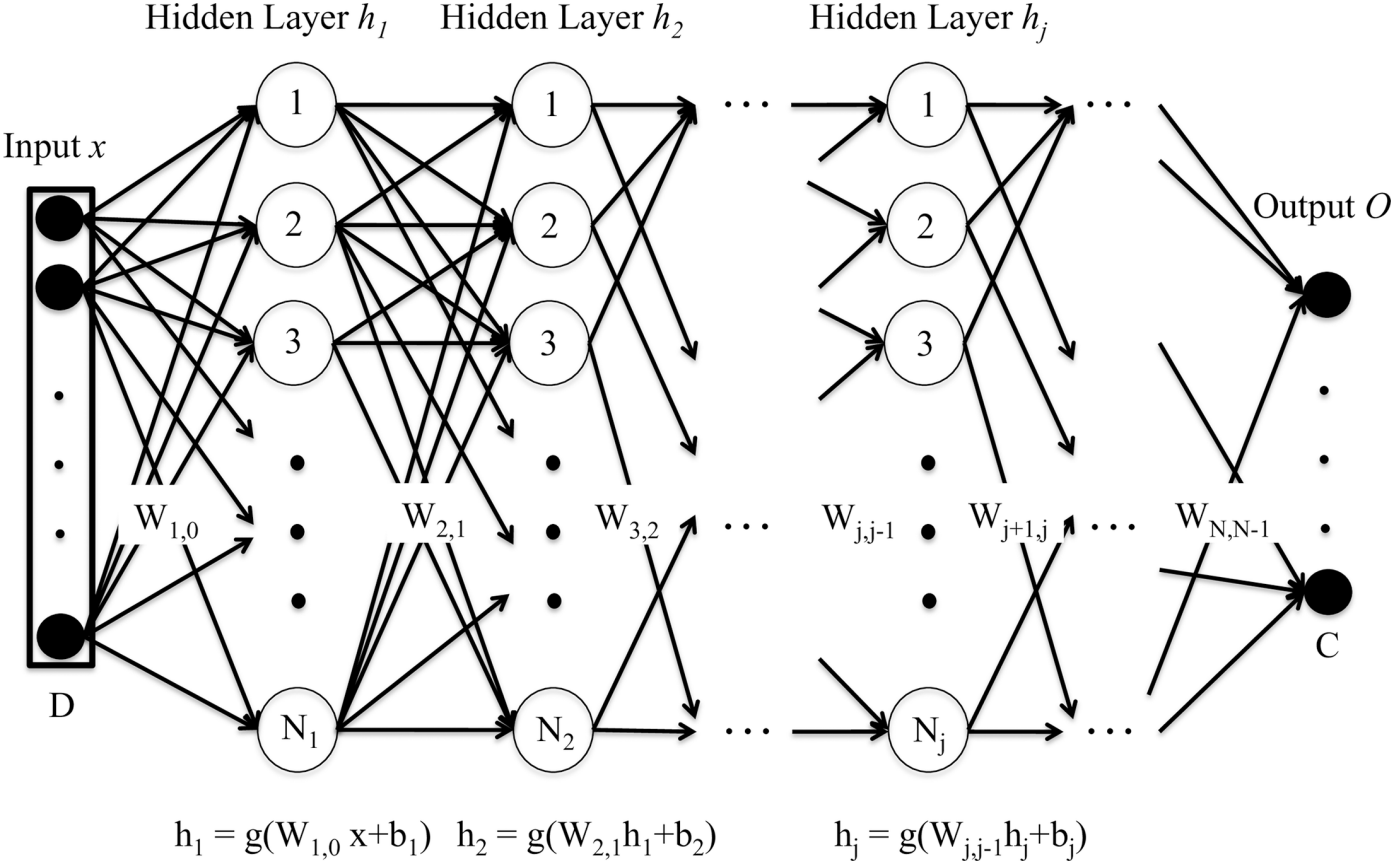
Deep neural network (DNN). This is a graphical representation of a standard feedforward DNN architecture. The DNN is fed with an input vector *x* of dimension D, which is transformed by the hidden layers *h*_*j*_ (composed of *N*_*j*_ hidden units) according to a function *g* and the parameters of the DNN (weights matrices *W* and bias vectors *b*). Finally, the output layer *O* provides the output of the DNN for the target task (for the case of classification, the probability of an input vector to belong to each class C).

Deep neural networks (DNN) are machine learning tools, which allow to learn complex non-linear functions of a given input in order to minimize an error cost. A graphical example of a standard deep neural network can be seen in Fig 1.

This way, a feedforward DNN used to perform a classification task might have the following general structure: an input layer, which is fed with some input vectors representing the data; two or more hidden layers (in opposition to shallow architectures, which had just one hidden layer), where a transformation is applied to the output of the previous layer, obtaining a higher level representation as we move away from the input layer; and an output layer, which computes the output of the DNN. In this last layer, the output is compared (for the case of supervised learning) to the reference label (true value) and the error criterion is applied to compute the cost.

The model is defined by its parameters: weight matrices *W*_*j*,*j*−1_ and bias vectors *b*_*j*_, with *j* going from 1 to the number of hidden layers. These parameters are adjusted iteratively to minimize a cost function, typically with stochastic gradient descent.

Thus, given a training set (*x*^(*i*)^, *y*^(*i*)^), where *x*^(*i*)^ is a given feature vector, and *y*^(*i*)^ its corresponding class (true value), each hidden layer applies a non-linear transformation function *g* to the output of the previous layer. This transformation takes into account the parameters *W* and *b* which relate one layer to its previous one, and provides the activation values of neurons with the following equations:

$$\begin{array}{c} h_{j}\left(x^{\left(i\right)}\right)=g\left(W_{j,j-1}h_{j-1}\left(x^{\left(i\right)}\right)+b_{j}\right),\;j=2,...,N-1 \end{array}$$ (1)

$$\begin{array}{c} h_{1}\left(x^{\left(i\right)}\right)=g\left(W_{0,1}x^{\left(i\right)}+b_{1}\right) \end{array}$$ (2)

Finally, for a classification task, the output layer computes a softmax function, which outputs the probability *P* of a given input *x* to belong to a certain class *c*:

$$\begin{array}{c} P\left(c|h\left(x\right)\right)=\frac{\exp(W_{l}^{c}h_{l}\left(x\right)+b_{l}^{c})}{\sum_{k=1}^{C}\exp\left(W_{l}^{k}h_{l}\left(x\right)+b_{l}^{k}\right)} \end{array}$$ (3)

where *h*_*l*_(*x*) refers to the last hidden layer activation for input *x*, $W_{l}^{c}$ and $b_{l}^{c}$ denote the weights matrix and bias vector receptively, which connect the output unit for class *c* with the last hidden layer, and *C* is the total number of classes.

In order to adjust the parameters to the task, a cost function is considered, trying to minimize the error between the prediction (output by the network) and the true class, and parameters are modified step by step via backpropagation [14].

### Applications to speech processing

Since the introduction of deep neural networks (DNN) in the field of speech processing, ASR systems have experienced a remarkable progress. The first successful applications were shown with the use of DNN based acoustic models in automatic speech recognition (ASR) systems [7], in which GMMs that compute the posterior probability of an input frame to correspond to a given phoneme, were replaced by DNNs in order to compute those phoneme posteriors.

Motivated by the outstanding results in ASR, DNNs were introduced in language and speaker recognition systems, following the same idea: replacing parts of the system with DNN based models. For instance, one of the systems used in [9] used a DNN trained for ASR instead of a GMM (UBM) model to compute posterior probabilities.

Some other trends applied different deep learning approaches to develop end-to-end systems for language and speaker recognition [15–17]. For language recognition, some successful applications based on DNN and LSTM-RNN (Long Short-Term Memory Recurrent Neural Networks) can be found in [15, 16], but still far from results obtained with bottleneck feature based approaches [13]. For speaker recognition this end-to-end scheme did not show the same success in terms of performance in comparison with the classical i-vector systems yet, especially in text-independent task (no restrictions on spoken content) with long utterances. However, for text-dependent speaker recognition tasks (typical in password based access control systems), end-to-end systems based on deep neural networks outperformed already the state-of-the-art in the field [17].

Nevertheless, the most successful DNN based approach for language and speaker recognition so far has been the use of a DNN for feature extraction [8, 18, 19]. This last approach will be the one followed in this work, applied to language recognition in acoustically similar languages. Then, we systematically explore how different configurations might influence the final LID performance and over the same dataset and framework.

## Bottleneck features for language recognition

**Fig 2:**
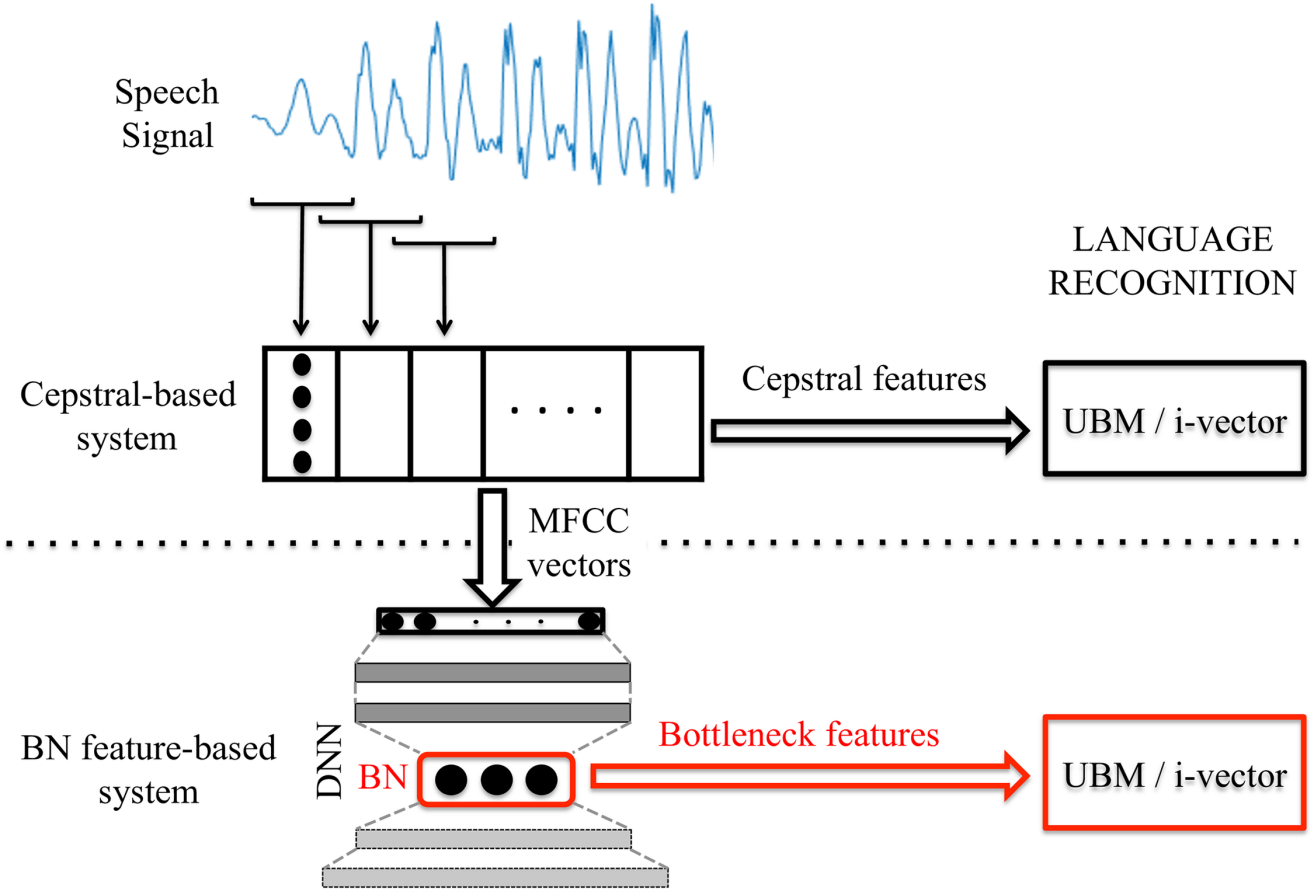
Representation of language recognition system structure. This is a graphical representation the language recognition systems, both the reference (cepstral feature based system) and the bottleneck feature based system.

As mentioned before, language recognition systems based on bottleneck (BN) features have become the state-of-the-art in the field. In these systems, a deep neural network (DNN) with a bottleneck layer (BN) is trained for ASR. This DNN is fed with input vectors representing frames of audio segments, and the time-dependent output of the bottleneck layer is used as a new frame-by-frame representation of the audio signal. With those feature vectors, the classical UBM/i-vector scheme is used to perform the language recognition task. A representation of this structure is shown in Fig 2.

In this section, we will describe these two steps: bottleneck feature extraction and i-vector language recognition system.

### Bottleneck features

**Fig 3:**
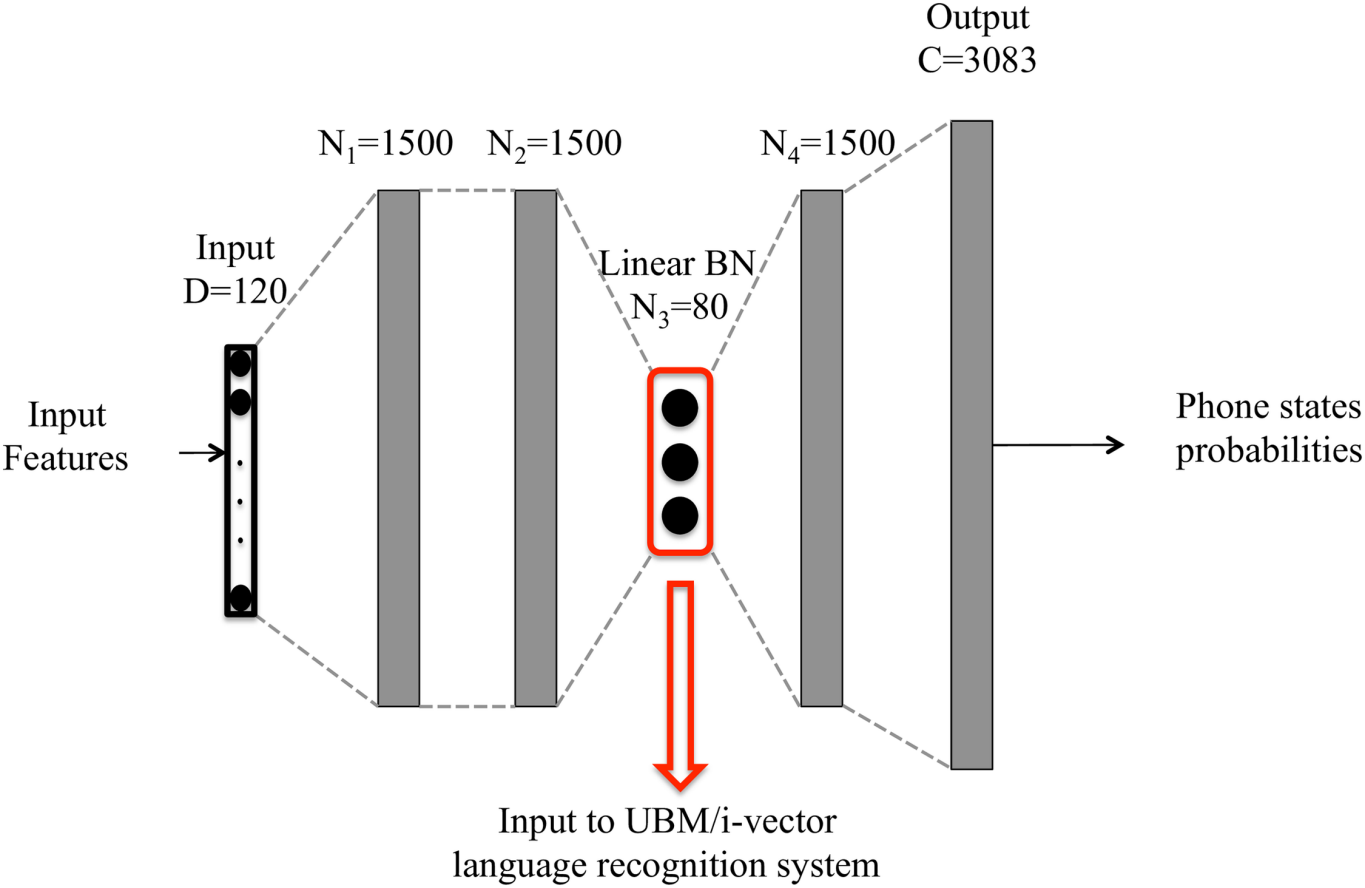
Example of DNN architecture with bottleneck layer. This is a graphical representation of the topology of a DNN with a BN layer, whose outputs (activation values) are used as input feature vectors for the language recognition system.

Broadly speaking, bottleneck features can be seen as a new representation of the frames of an audio signal, learnt directly by a DNN. The underlying motivation is to obtain more abstract feature vectors, which help to model the feature space and contain useful information, allowing the network to learn it by itself and reducing the dependency of hand-crafted features. An example of the DNN structure with a bottleneck layer used in this work can be seen in Fig 3.

A DNN with a bottleneck layer is a feed-forward neural network with several hidden layers where one of them is relatively small with respect to the rest. Moreover, it usually applies a linear transformation in contrast to non-linear transformations applied in the rest of the hidden layers. This bottleneck layer aims to compress the information learnt by the previous layers, projecting into an useful representation for the task for which the DNN is trained (for instance, automatic speech recognition) [20]. Even though the bottleneck layer forces the network to compress information making the classification harder (as we mention in Section Number of Hidden Layers), it has advantages as the transformation from input to classification is less straightforward making the system less prone to overfitting and more robust [13], and it achieves dimensionality reduction from a full hidden layer in order to be used as feature vectors for other tasks.

In this work, and typically in language recognition systems based on bottleneck features, the DNN is trained for ASR. This is motivated by the phonemes and phoneme sequences being different depending on the language. In fact, the first successful approaches to automatic language recognition were systems based on phonetic information (PRLM, PPRLM) [21]. Therefore, the phonetic information has proved to be useful for the task of language recognition itself [11, 13].

In particular, the DNN used in this work is a frame-by-frame classifier for triphone states (i.e. different states of a context-dependent phoneme model). The architecture consists of an input layer, a number of hidden layers which apply simple non-linear functions (as *sigmoid* or *tanh* functions), and an output layer. This last layer applies a *softmax* function in order to provide the probabilities of a frame representing each triphone state. The DNN in this work is trained with English utterances (Switchboard database) and there are thousands of triphone states in that language. However, in practice, there are not enough samples from each of these states to properly train ASR systems. Moreover, some works (such as [11, 18]) showed that taking into account a subset of the total number of combinations of triphone states as targets, is enough to obtain good performance in the DNN used to extract bottleneck features for other tasks. Thus, we use a subset of 3083 outputs for the DNN, which are the most probable triphone states found while training the ASR system.

### Language recognition: The i-vector approach

Total Variability modeling and the corresponding i-vector approaches have been the state-of-the-art in speaker recognition for several years [4]. Given the success this technique showed in that field, it was adapted and introduced in the language recognition research community [5], becoming the state-of-the-art in this area as well.

In a classical i-vector pipeline, we can split the system into different steps:

- UBM-GMM modeling
  The first step of this approach to language recognition consists in modeling the feature space with a Gaussian Mixture Model (GMM). This GMM is trained from feature vectors (as for example, MFCC or bottleneck features, in our case), with the Expectation Maximization (EM) algorithm, with data from a great number of utterances that belong to different languages. The resulting GMM is known as Universal Background Model (UBM), and is defined by its mean vector (*μ*, concatenation of mean vectors of each Gaussian component known as *supervector*) and its covariance matrix (Σ, covariance matrices of each Gaussian component).
- Statistics computation
  Given a trained UBM, defined by its parameters *λ* = {*μ*, Σ}, the next step is to compute the Baum-Welch statistics for a given utterance. These statistics represent each frame according to the GMM-UBM. Then, for each Gaussian component *c*, and each utterance frame *u*_*t*_, the zero- and first-order sufficient statistics are obtained as follows:
  $$\begin{array}{c} N_{c}=\sum_{t}P(c|u_{t},\lambda ) \end{array}$$ (4)
  $$\begin{array}{c} F_{c}=\sum_{t}P(c|u_{t},\lambda )\left(u_{t}-\mu \right) \end{array}$$ (5)
  where *P*(*c*|*u*_*t*_, *λ*) is the posterior probability of component *c* generating the frame *u*_*t*_.
- Total variability subspace training and i-vector extraction
  Generally speaking, the idea of the Total Variability (TV) approach is to project the supervector of means from a given utterance into a subspace *T* in which the variability (both channel and language) of a training dataset is represented [4].
  The *T* projection matrix is trained via Expectation Maximization (EM), with a dataset which includes variability useful for the target task (language variability, for the language recognition case).
  Then, the total variability model can be represented as follows:
  $$\begin{array}{c} \mu_{UTT}=\mu_{UBM}+Tw \end{array}$$ (6)
  where *μ*_*UTT*_ is the utterance-dependent supervector, *μ*_*UBM*_ is the UBM supervector of means (language-independent) and *w* is a latent variable, whose point estimated with Maximum A Posteriori (MAP) will be the i-vector representing each utterance.
  To extract the i-vector corresponding to a given utterance, the UBM is used to collect the Baum-Welch statistics from the utterance. Once these statistics and the *T* matrix are available, each i-vector can be extracted with the following formula [4]:
  $$\begin{array}{c} w={\left(I+T^{t}\Sigma^{-1}NT\right)}^{-1}T^{t}\Sigma^{-1}F \end{array}$$ (7)
  where *N* and *F* are matrices composed of the zero- and first-order statistics, and Σ is the covariance matrix of *F*. These i-vectors will have information of the language contained in the utterance they represent, since that is the task for which the *T* matrix has been trained.
- Classification
  Finally, once the i-vectors are computed, classification is performed. One of the basic approaches is the cosine distance scoring, in which a score is extracted for each trial or comparison between test and train (language model) i-vectors. The higher the resulting score is, the higher is the probability to belong to the same class, since those data points are closer in the i-vector space. This will be the classifier considered in this work.
  In the field of speaker recognition, some other scoring approaches in the i-vector subspace are usually applied to compensate the variability still existing in the i-vector such as Linear Discriminant Analysis (LDA) or Probabilistic LDA (PLDA). However, these approaches were not successfully applied to language recognition, due to the projection into a (N-1)-dimensional space (where N is the number of languages involved in the task) with the consequent loss of information for LID, where the number of classes is much smaller than in the case of speaker recognition [5].

## Experimental framework

### Database description: Switchboard and NIST LRE 2015

#### Training datasets

The language recognition system used in this work has two clearly separated parts to be trained: the DNN used as feature extractor, and the i-vector pipeline (which includes GMM-UBM and total variability subspace training).

Thereby, two different databases are considered in order to train each part: Switchboard database and NIST LRE 2015 training data.

- Switchboard
  In this work, we use the Switchboard (Part 1 release) database [22] to train the DNN to be used later as a bottleneck extractor. This set contains approximately 320 hours of speech (telephone conversations in English) from around 4800 speakers. A 10% of this dataset is reserved to validate the DNN performance.
  This dataset is labeled for speech recognition purposes, at word level, and will be used to train an ASR system (developed in Kaldi [23], a toolkit for speech recognition widely used in the field) to obtain triphone state level label alignments. These alignments are then used to train the DNN with a bottleneck layer.
- NIST LRE 2015 training data
  To train the language recognition system (UBM and *T* matrix), we use the NIST Language Recognition Evaluation 2015 training data [24].
  As in other evaluations, the dataset contains both conversational telephone speech (CTS) and broadcast narrowband speech (BNBS) data. It involves segments of speech from twenty different languages, grouped into six clusters according to similarities and relation between languages (see Table 1). This way, the focus of this evaluation is distinguishing among closely related languages, i.e., within each cluster.
  In particular, we use the data provided for the core task (with limited data) of the NIST LRE 2015, which contains a set of segments from the twenty target languages. The segments were audited by the Linguistic Data Consortium (LDC), so they contain at least 30 seconds of speech belonging to the labeled language. It should be taken into account that the amount of data per language, even within a cluster, varies notably. For example, the English cluster includes about 30 minutes of British English but, however, more than 100 hours of General American (see Table 1).
  To train the i-vector language recognition system we choose randomly 85% of this training data, using the remaining 15% for evaluation purposes as explained below.

**Table 1:** Cluster of target languages and approximate amount of data per language in the NIST LRE 2015 training dataset.

Cluster	Target Languages	Hours of data
Arabic	Egyptian	95.4
	Iraqi	37.2
	Levantine	41.1
	Maghrebi	38.6
	Modern Standard	3.7
Chinese	Cantonese	3.4
	Mandarin	71.8
	Min	8.1
	Wu	7.7
English	British	0.5
	General American	100.0
	Indian	8.1
French	West African	7.7
	Haitian Creole	2.7
Slavic	Polish	30.0
	Russian	18.0
Iberian	Caribbean Spanish	26.9
	European Spanish	8.1
	Latin American Spanish	6.9
	Brazilian Portuguese	0.8

#### Test datasets

In this work, we evaluate our language recognition systems in two different datasets from the NIST LRE 2015.

- Matched test dataset
  Firstly, we consider a matched setup, in which we evaluate the systems with the 15% of the training NIST LRE 2015 dataset which has not been used for training the language recognition system. We segmented the speech recordings into fragments of 3, 10 and 30 seconds of speech. The number of fragments used for each subset is 84446, 25592 and 8757, respectively. This corresponds to approximately 70 hours of actual speech for this matched test dataset.
- Mismatched test dataset
  Then, in order to test how the reference system and the developed bottleneck features based system perform in a mismatched dataset (from a totally different collection of audio recordings with respect to data used to train the systems), we present this comparison over the evaluation data of NIST LRE 2015. In this case, test segments are not constrained to have a specific duration as in previous evaluations and in our other test set (3, 10 or 30 seconds). Instead, they covered a broad range of durations, from 3 to 260 seconds of speech. The evaluation dataset includes 164334 segments from all the target languages used in training, both from CTS and BNBS. More details can be found in the LRE’15 evaluation plan [24].

This structure of subsets for training and testing the systems is summarized in Table 2.

**Table 2:** Datasets used for training and testing our systems.

	DNN	UBM/i-vector
Train	Switchboard (90%)	LRE’15 (85% from training data)
Test	Switchboard (10%)	1) Matched dataset (15% from training data, LRE’15) 2) Mismatched dataset (evaluation data, LRE’15)

### Evaluation metrics

In order to evaluate the developed systems, we used different evaluation metrics.

Firstly, we show results of the DNNs used as bottleneck feature extractors in terms of phoneme state frame accuracy, i.e. the percentage of frames classified correctly by the DNN according to the given phoneme state labels.

Secondly, performance for the final language recognition task is presented as average Equal Error Rate (EER): we first compute EER as a one-versus-all approach, and then average them to have a final average EER. Since we are evaluating in the context of LRE 2015, which involves clusters of similar languages, we treated each cluster separately as it was done in the evaluation, not taking into account scores of a given test segment outside the cluster it belongs to. This way, we compute the average EER for each cluster, and use the final average of those partial results as the final language recognition system performance.

### Cepstral based i-vector Reference System Description

The reference language recognition system considered in this work follows the classical i-vector based approach described in Section Language Recognition: the i-vector approach, in which each i-vector will be a low-dimension representation of a given utterance.

In order to compute the mentioned i-vectors, each audio recording is represented by a feature vector for each frame (or segment of 20 ms of speech in our system). We use the Mel-frequency Cepstral Coefficients (MFCC) as parameters (or input feature vectors) for our reference system. These parameters represent the acoustic information contained in the audio recordings. In particular, in this work, we augmented the MFCCs with temporal information given by the Shifted Delta Cepstral coefficients (SDC) [25], in order to consider the information given by the context of a certain frame.

To compute these input vectors, we use a Hamming window of 20 ms, with 50% overlap, and a filter-bank of 25 Mel-scaled filters. We compute then the MFCC-SDC for each frame [25], created by stacking delta cepstra coefficients computed across multiple speech frames. In particular, we use 7 MFCCs, with one frame advance and delay for the delta computation, and we stack 7 blocks with a time shift of 3 frames between them (7-1-3-7 configuration), which results in 56-dimensional feature vectors representing each frame of the utterance.

In this feature space, an Universal Background Model (UBM) composed of 1024 Gaussian components is trained, and Baum-Welch statistics are computed over this UBM for each utterance. Then, a Total Variability (TV) subspace of 400 dimension is derived from them by using PCA (Principal Component Analysis) followed by 10 EM iterations. All this process is carried out using Kaldi [23]. Finally, cosine similarity is used to classify the resulting i-vectors. Results of this system can be seen in Table 3.

**Table 3:** Cepstral based i-vector reference system (i-vector based on MFCC-SDC features) performance, average EER of all language clusters.

Duration	30s	10s	3s
**EER (in %)**	7.35	14.08	21.56

The performance of our reference system improves with the amount of speech contained in the test segments. This way, results range from 7.35% of average EER in the case of test segments containing 30 seconds of speech to 21.56% for shorter segments of 3 seconds. This is due to a better estimation of the i-vector obtained when the amount of speech is enough to have reliable estimations for this approach. It should be noticed that the target task is classification among similar languages, in the context of the LRE 2015, which makes the task especially difficult when test segments are short (few seconds of speech).

### Bottleneck feature based language recognition system description

To develop the language recognition system used in this work, frame level bottleneck features are extracted from a DNN trained for ASR. This bottleneck feature vectors replace the MFCC-SDC feature vectors used in the reference system.

The DNN architecture used in this work is a feedforward network with an input layer, three to five hidden layers, and the output layer.

To feed the network, 20-dimensional MFCC input vectors are used and preprocessed stacking 31 frames together (15 frames of context in both temporal directions). Then, the temporal trajectory of each MFCC coefficient is smoothed by applying a Hamming window followed by a DCT of which only coefficients 0 to 5 are kept, as done in [26]. The resulting 120-dimensional feature vector is used as the input to the DNN.

This input layer is followed by three to five hidden layers, which perform non-linear transformations (except the bottleneck layer) of the input in order to obtain a better phoneme state classification performance. These hidden layers are composed of 1500 units which apply the *sigmoid* function as activation or non-linear transformation. The activation value computed in each unit is the resulting value of the application of the non-linear function to the input multiplied by the weights and bias.

The bottleneck layer applies a linear transformation, and we vary its size and position depending on the experiment.

Finally, the architecture is completed with a softmax layer, which outputs the probability of each input to correspond to a given phoneme state. In our case, the output layer tries to discriminate among 3083 triphone states.

The training algorithm used is the stochastic gradient descent with batches of size 512 samples (i.e. computing the gradients with each block of 512 input segments), and the cost function we aim to optimize is cross-entropy. The training process is stopped when the error function converges according to a validation set, a disjoint set from the training data (the remaining 10% of Switchboard dataset not used for training).

## Experiments and results

In order to develop a bottleneck feature based language recognition system, many parameters have to be tuned. In this work, we first obtain the phonetic alignments of a given set of Switchboard with an ASR system via Kaldi with a fixed configuration. The configuration parameters of the UBM/i-vector system used as language recognition backend are also fixed through the experiments in this work. Then, we explore different configurations of the DNN used as bottleneck feature extractor. Thus, in this section, we describe these experiments varying the DNN architecture and present results, both in terms of DNN performance (frame accuracy of phoneme states classification) and final language recognition performance (average EER), as it was described in Section Evaluation Metrics.

We evaluate the language recognition system in the test-development dataset described in Section Test Datasets, where we explore the influence of variations in the topology of the DNN, and, finally, we show the results in the evaluation dataset of the LRE 2015.

### Results on the matched test dataset

#### Number of hidden layers

This first set of experiments aims to evaluate the performance of systems in which the number of hidden layers in the DNN varies from 3 to 5, with the bottleneck layer occupying central positions (layers 2, 3 and 3, respectively). In spite of resulting in a better performance in terms of phoneme frame classification with the 5 layers configuration (see Table 4), it is the architecture with 4 hidden layers the one that reaches the lowest EER in terms of language recognition performance (see Table 4).

**Table 4:** DNN (phoneme classification, frame accuracy) and language recognition performance (average EER of all language clusters).

Number of Hidden Layers	DNN	EER (in %)		
	Frame Accuracy	30s	10s	3s
3	47.82	5.52	9.04	14.34
4	49.55	**4.33**	**7.81**	**13.76**
5	**50.46**	5.22	8.57	14.15

**Fig 4:**
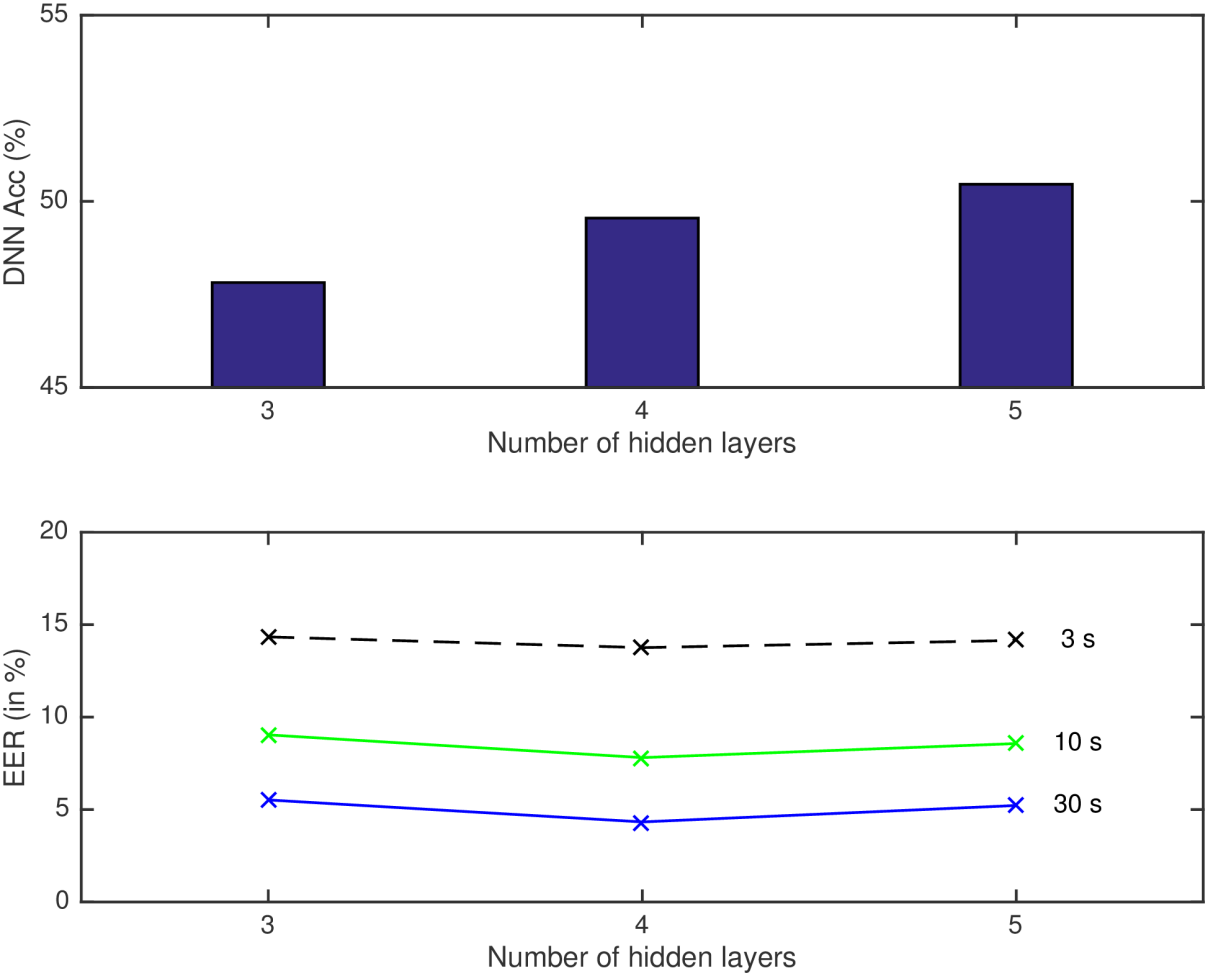
Phoneme frame accuracy of DNN (upper part of the figure) and language recognition systems (lower part) for different test durations (3, 10 and 30s) with different number of hidden layers of the DNN.

It is very interesting to see that the system which gives the best performance in terms of phoneme frame accuracy does not lead to a better bottleneck feature extractor for LID (see also Fig 4). This might be partially because the ideal speech recognizer would suppress as much as possible information not important for phoneme discrimination, which might include relevant information to distinguish between languages. Also, there is a possible overfitting to the database used to train the DNN, which is different from the one where the LID task is evaluated. As we have mentioned before, phonetic information is useful for the task of language recognition, since phonemes and phoneme sequences vary depending on the language. However, although both tasks ASR (for which the DNN is trained) and LID might share some information, it is not exactly the same since more factors are involved, and this is related to the difference in trends of phoneme frame accuracy and LID performance.

Also, a DNN with a bottleneck layer can be seen as two different networks, which are focused on different tasks: the first network would be the complete network trained for ASR; and the second network would be the part from the input to the BN layer, which can be used as feature extractor for other tasks (LID in our case). Thus, for the complete network, the BN layer imposes a constraint for the information to pass through the network. Such restriction is damaging the performance of the network in terms of frame accuracy for ASR: the same networks trained with three and four full hidden layers (1500 hidden units as the rest) reached a frame accuracy of 49.29% and 50.08% respectively, performance that drops when including the BN layer to 47.82% and 49.55% respectively, as we can see in Table 4. However, that information compression is good if the aim is to use that restricted information for other task as LID. Therefore, for the LID system used in this work, we want the DNN to focus mainly on the second part, to obtain a compact representation of the signal useful for LID and not optimize the DNN only for ASR. Therefore, the discriminative task (ASR) is easier for the DNN when the classifier is more complex (5 layers DNN), which is making easier the classification task, and, thus, improves the frame accuracy. However, that network is not being forced to focus on obtaining a compact representation of the signal (and hopefully good), which is used for LID afterwards. Even though there might be other factors influencing this, these explanations support the results where not the best frame accuracy of the DNN related to the task of ASR leads to the best DNN as feature extractor for LID.

#### Bottleneck layer position

Keeping fixed the architecture of the DNN to four hidden layers, we explore how the language recognition system performs depending on the position that the bottleneck layer occupies in the network. Results can be seen in Table 5.

**Table 5:** DNN (phoneme classification, frame accuracy) and language recognition performance (average EER of all language clusters).

Position of BN Layer	DNN	EER (in %)		
	Frame Accuracy	30s	10s	3s
First	49.17	9.37	12.24	16.59
Second	49.46	6.27	9.55	14.58
Third	**49.55**	**4.33**	**7.81**	**13.76**
Fourth	48.05	4.64	8.00	14.17

These experiments were carried out in order to explore how different layers of the DNN, which correspond to different levels of extracted information, perform in terms of language recognition. Feedforward DNNs are trained to automatically learn a transformation that maps an input to an output. Each of the hidden layers is then learning features which help with that discriminative task, and as it has been seen in different research works [12], deeper layers learn more abstract features, with more information which help to final classification. In this case, the position of the BN layer is related to the degree of proximity of the information to the phonetic states that the network learns to code.

The closer the BN layer to the input layer, the noisier the resulting representation would be, which might explain the drop in performance for the first and second layers with respect to the results of the last two layers.

**Fig 5:**
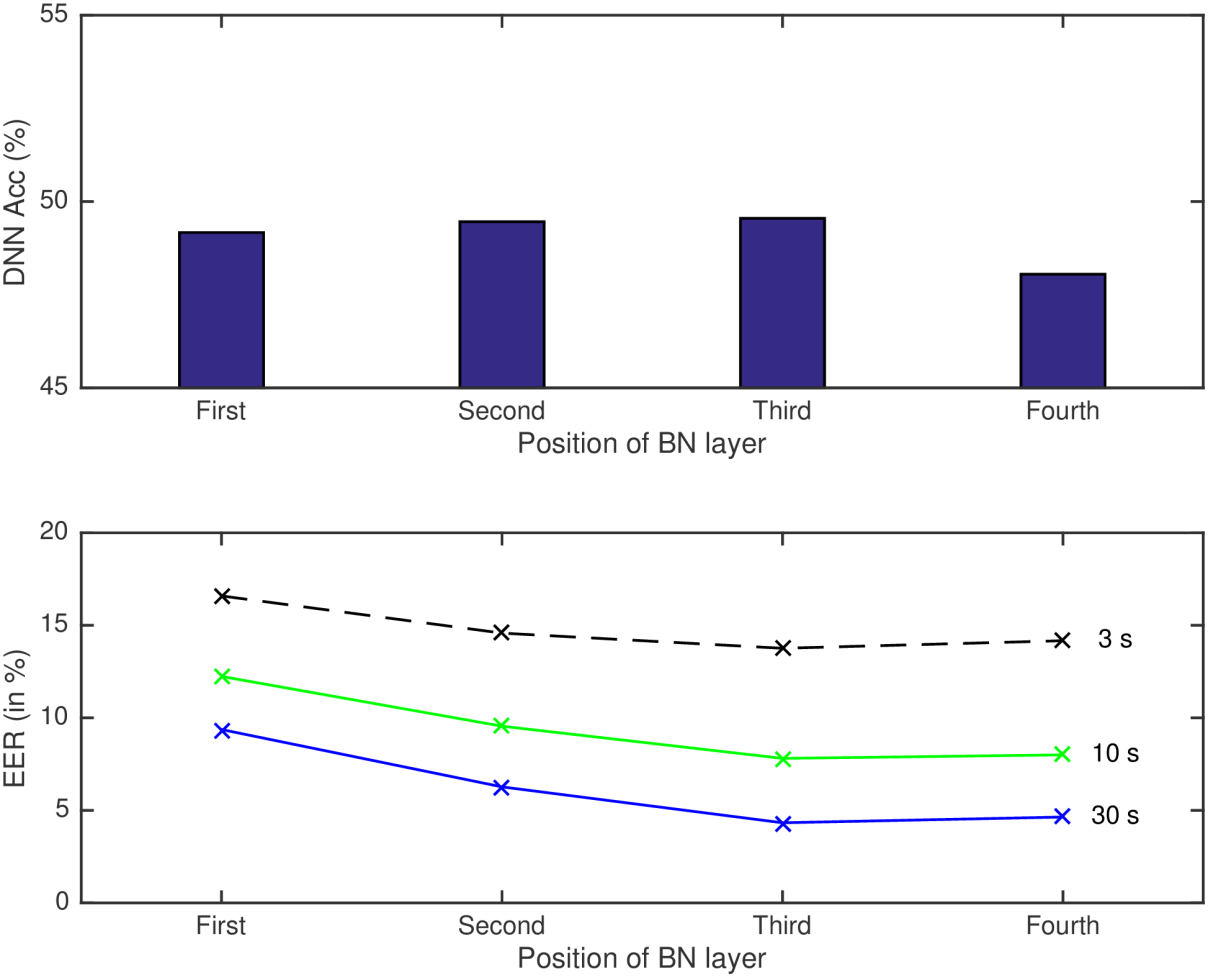
Phoneme frame accuracy of DNN (upper part of the figure) and language recognition systems (lower part) for different test durations (3, 10 and 30s) when the bottleneck layer moves from first to fourth layer in a four hidden layer topology.

The best performance in terms of EER for language recognition is obtained when the bottleneck layer is located in the third layer, but that result is very close to the one obtained with the BN at the fourth layer, as it can be seen at the bottom side of Fig 5. Bottleneck features from these two layers seem to contain useful information, although still third layer, which is further from the output layer, gives the best results.

Performance of the DNN for phoneme state classification also drops when the BN layer moves from layer third to fourth, from 49.55% to 48.05%. In this topology, the bottleneck layer in position fourth is connected directly to the output layer, resulting in a weight matrix that connects a small layer with just 80 hidden units with the output layer, of size 3083. These weights might be difficult to learn, which may explain this drop in performance of the DNN.

#### Bottleneck layer size

This set of experiments focuses on the size of the bottleneck layer, i.e. the number of hidden units in the bottleneck layer. We train six different neural networks with sizes of the bottleneck layer ranging from 20 to 120 with a step of 20 units. Results are shown in Table 6.

**Table 6:** DNN (phoneme classification, frame accuracy) and language recognition performance (average EER of all language clusters).

Size of BN Layer	DNN	EER (in %)		
	Frame Accuracy	30s	10s	3s
20	46.60	6.42	11.73	18.58
40	48.81	4.57	8.67	15.07
60	49.20	4.63	8.27	14.33
80	49.55	**4.33**	**7.81**	13.76
100	49.60	4.51	7.82	13.39
120	**49.70**	4.94	7.99	**13.08**

**Fig 6:**
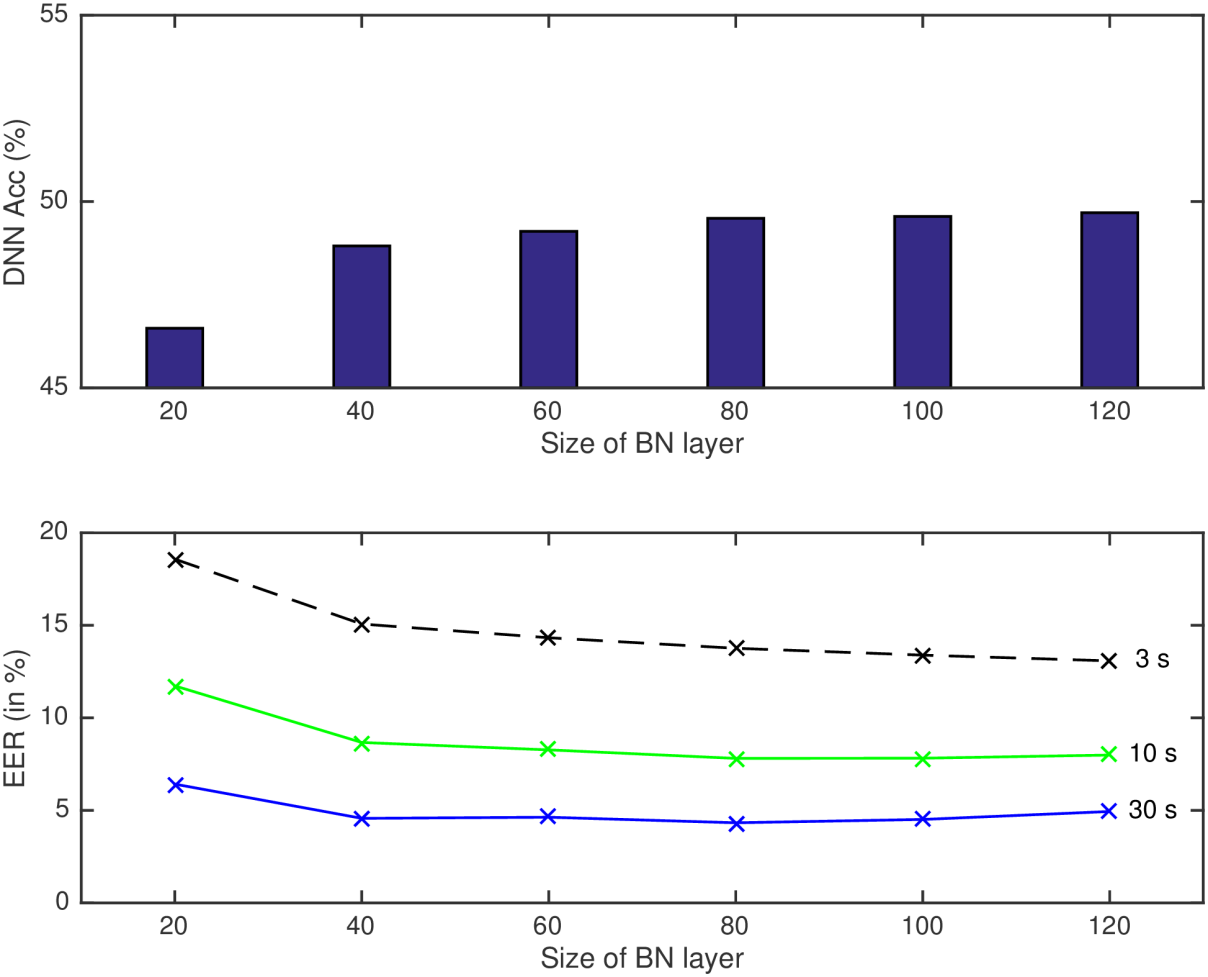
Phoneme frame accuracy of DNN (upper part of the figure) and language recognition systems (lower part) for different test durations (3, 10 and 30s) when the bottleneck layer size (number of hidden units) varies.

In this case, we observe a big gap in performance between the 20 and the 40-dimensional bottleneck layers, both in terms of DNN classification accuracy and language recognition performance (see the elbow at size of 40 units for the bottleneck layer in Fig 6). The rest of the sizes seems to perform in a similar way, with relative improvements not bigger than 9% relative for 30 seconds long utterances, and around 13% relative for test segments of 3 seconds of duration.

As we can see from the results, frame accuracy increases with the size of the bottleneck layer. In fact, the constraint for the information to flow through the network imposed by the bottleneck layer is not beneficial for ASR itself (as we mentioned in Section Number of Hidden Layers with the improvement of frame accuracy when training the DNN with full hidden layers. However, that restriction of information included with the BN layer is useful for the use of the DNN as feature extractor for the purposes of LID and other tasks. At the same time, large BN feature vectors increase the complexity of the UBM/i-vector system, which has to deal with high dimensional spaces. As it was shown with the introduction of total variability in the field, which allowed to reduce the feature space dimensionality from the supervector to the i-vector, modeling on low dimensional spaces makes the task less difficult.

Nonetheless, it should be noted that the larger the bottleneck, the more memory resources and computation time is needed to train the language recognition system, due to the increase in the dimensionality of the feature space.

For these reasons, we select as the best configuration the one with a 80-dimensional bottleneck layer for this setup. It can be observed that the best performance in terms of language recognition for the case of 3 seconds segments is obtained when bottleneck feature vectors have a dimension of 120, showing that for very short speech segments the system might benefit from using larger bottleneck layers.

### Results on the mismatched test dataset

In this section, we show the results over the mismatched test dataset considered in this work, which corresponds to the evaluation dataset provided by NIST for the LRE 2015.

**Fig 7:**
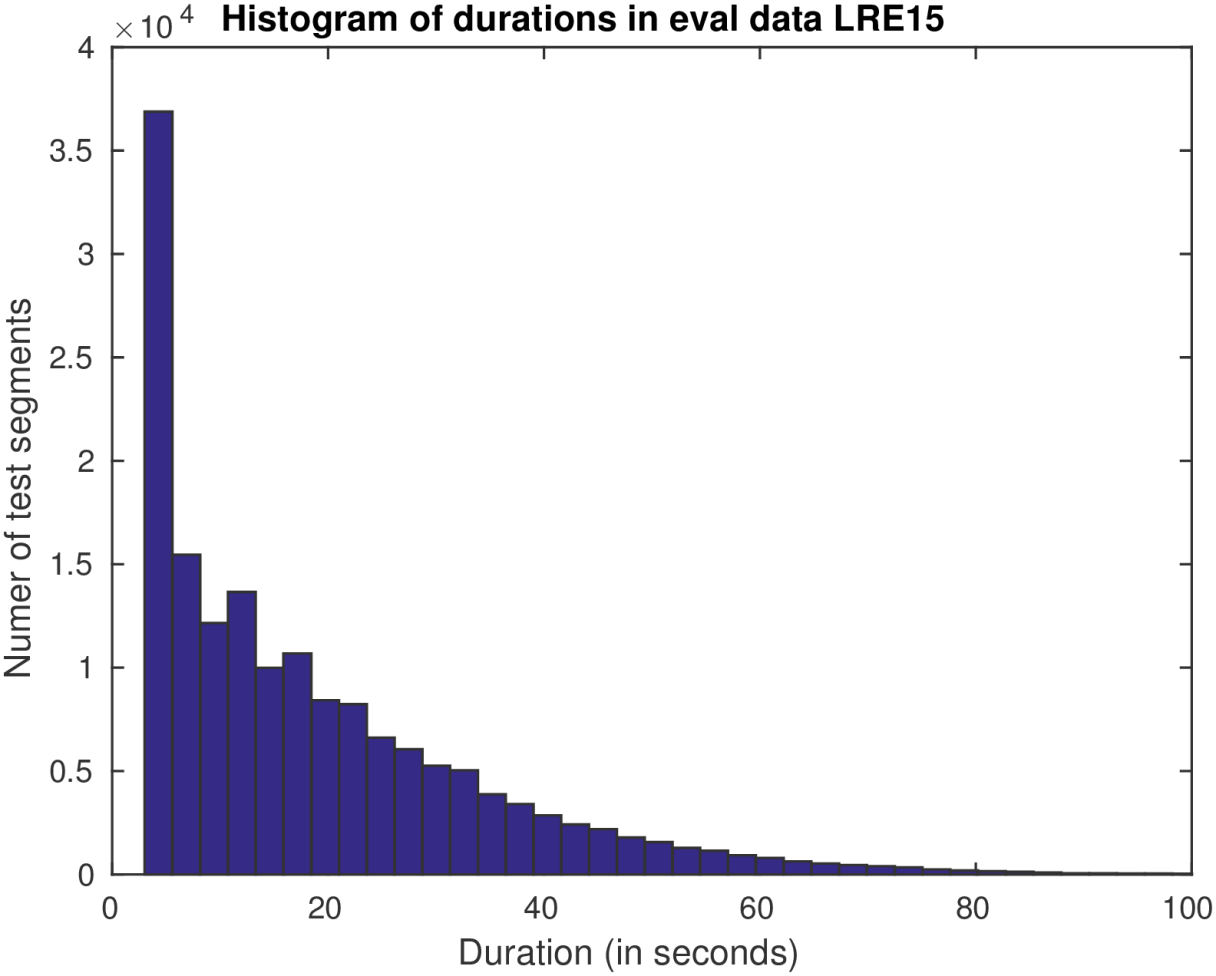
Test duration segments histogram of the mismatched test dataset (the evaluation data of LRE 2015).

Different challenging aspects were involved in this evaluation: it focused on similar languages divided in clusters, and, also, on short test segments evaluated as a single task. The distribution of durations of test segments is shown in Fig 7.

**Fig 8:**
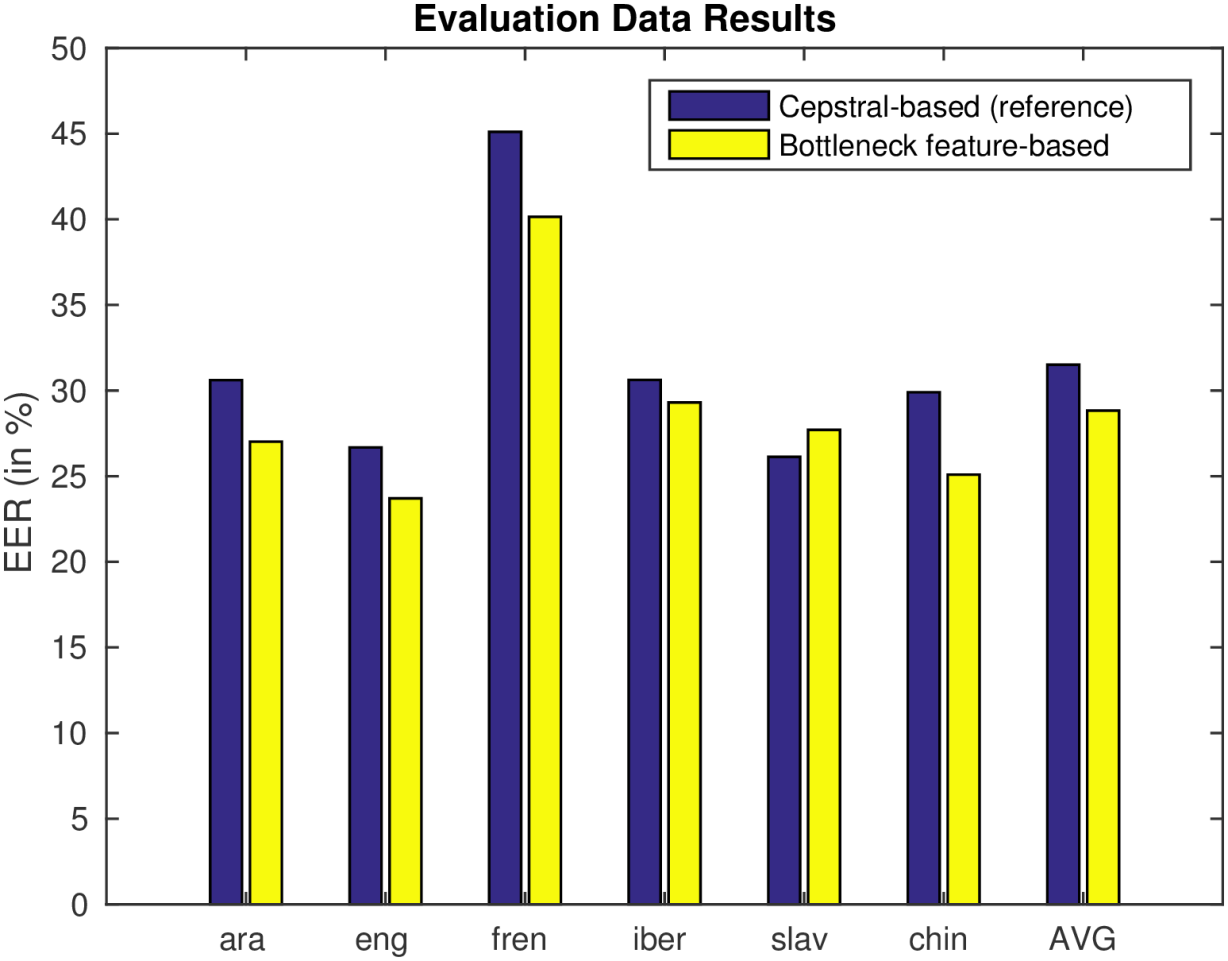
Evaluation data results. This figure shows the performance per cluster and on average for the cepstral based i-vector reference system and the bottleneck feature based language recognition system, for the best configuration found on the development and over the actual evaluation data of LRE’15. This configuration was 80-dimensional bottleneck features from third hidden layer in a four hidden layer DNN architecture.

We evaluate both the reference cepstral based i-vector system and the best configuration according to development results, separated by cluster and on average for all of them (see Table 7 and Fig 8, respectively). The bottleneck based approach shows a relative improvement of ∼8.5% in terms of average EER with respect to the cepstral based i-vector reference system based on MFCC-SDC features.

**Table 7:** Language recognition performance (average EER of all clusters) for the evaluation data of NIST LRE 2015.

System	EER (in %)
Cepstral-based (reference)	31.51
Bottleneck feature-based	**28.83**

Results over this test data (see Fig 8) show a degradation in comparison with relative improvements obtained in the development set. However, bottleneck feature-based approach outperforms the reference system based on MFCC-SDC features.

It should be noted as well that many factors are different between the development part used as test and this evaluation dataset, such as duration of test segments, or the mismatch existing between the train and test data in this case. Then, degradation in this test set might be mainly caused by the existing mismatch between training and evaluation data.

## Conclusions

In this work, we try to fill in the gap existing in research about the influence of the configuration of the deep neural network (DNN) used as a bottleneck feature extractor in a language recognition system, taking into account that variations in the topology of the DNN have not been analyzed in previous published works over the same framework. Thus, we explore different configurations: we present results varying the number of hidden layers, the position of the bottleneck layer in the DNN and the size of it, studying the effect of different representations obtained by the network both in terms of phoneme state frame accuracy of the DNN and language recognition EER. All these configurations are evaluated over the LRE’15 dataset.

We see that the performance of the DNN in terms of phoneme state classification do not correspond with the best performance of the resulting bottleneck features for language identification in the i-vector pipeline. This is caused by a combination of effects. Firstly, the better the DNN classifies the phoneme states, ideally, the more language information is dropped from the resulting bottleneck representation. Moreover, the database used to train and evaluate DNN performance is similar while the database used to evaluate LID performance is different to those used to train and evaluate DNN performance, which influences as well the differences in tendencies of performance of DNN and language recognition systems.

Finally, this bottleneck feature based language recognition system is compared to a reference system, following the same i-vector backend but based on MFCC-SDC parameterization of the input audio signal. This bottleneck feature approach shows relative improvements of about 36% for 3 seconds test segments, 44% for 10 seconds long segments and 41% when testing in 30 seconds fragments (increasing with duration), with respect to the reference system for the case of the test-development dataset (matched conditions). When testing this approach on the evaluation data of the NIST LRE 2015 (mismatched conditions), a degradation in relative improvement of the bottleneck features replacing the traditional parameters is observed, caused mainly by the mismatch existing between training and test data in this case. However, bottleneck feature based language recognition approach still outperforms the classical approach based on acoustic features (MFCC-SDC) used as reference in this work, with a relative improvement of approximately 8% on average.
